# Co-Crystal Structures of PKG Iβ (92–227) with cGMP and cAMP Reveal the Molecular Details of Cyclic-Nucleotide Binding

**DOI:** 10.1371/journal.pone.0018413

**Published:** 2011-04-19

**Authors:** Jeong Joo Kim, Darren E. Casteel, Gilbert Huang, Taek Hun Kwon, Ronnie Kuo Ren, Peter Zwart, Jeffrey J. Headd, Nicholas Gene Brown, Dar-Chone Chow, Timothy Palzkill, Choel Kim

**Affiliations:** 1 Department of Pharmacology, Baylor College of Medicine, Houston, Texas, United States of America; 2 Department of Medicine, University of California San Diego, La Jolla, California, United States of America; 3 The Verna and Marrs McLean Department of Biochemistry and Molecular Biology, Baylor College of Medicine, Houston, Texas, United States of America; 4 Rice University, Houston, Texas, United States of America; 5 The Berkeley Center for Structural Biology, Lawrence Berkeley National Laboratory, Berkeley, California, United States of America; 6 Department of Molecular Virology and Microbiology, Baylor College of Medicine, Houston, Texas, United States of America; 7 Computational Crystallography Initiative, Lawrence Berkeley National Laboratory, Berkeley, California, United States of America; University of Oldenburg, Germany

## Abstract

**Background:**

Cyclic GMP-dependent protein kinases (PKGs) are central mediators of the NO-cGMP signaling pathway and phosphorylate downstream substrates that are crucial for regulating smooth muscle tone, platelet activation, nociception and memory formation. As one of the main receptors for cGMP, PKGs mediate most of the effects of cGMP elevating drugs, such as nitric oxide-releasing agents and phosphodiesterase inhibitors which are used for the treatment of angina pectoris and erectile dysfunction, respectively.

**Methodology/Principal Findings:**

We have investigated the mechanism of cyclic nucleotide binding to PKG by determining crystal structures of the amino-terminal cyclic nucleotide-binding domain (CNBD-A) of human PKG I bound to either cGMP or cAMP. We also determined the structure of CNBD-A in the absence of bound nucleotide. The crystal structures of CNBD-A with bound cAMP or cGMP reveal that cAMP binds in either *syn* or *anti* configurations whereas cGMP binds only in a *syn* configuration, with a conserved threonine residue anchoring both cyclic phosphate and guanine moieties. The structure of CNBD-A in the absence of bound cyclic nucleotide was similar to that of the cyclic nucleotide bound structures. Surprisingly, isothermal titration calorimetry experiments demonstrated that CNBD-A binds both cGMP and cAMP with a relatively high affinity, showing an approximately two-fold preference for cGMP.

**Conclusions/Significance:**

Our findings suggest that CNBD-A binds cGMP in the *syn* conformation through its interaction with Thr193 and an unusual cis-peptide forming residues Leu172 and Cys173. Although these studies provide the first structural insights into cyclic nucleotide binding to PKG, our ITC results show only a two-fold preference for cGMP, indicating that other domains are required for the previously reported cyclic nucleotide selectivity.

## Introduction

**Figure 1:**
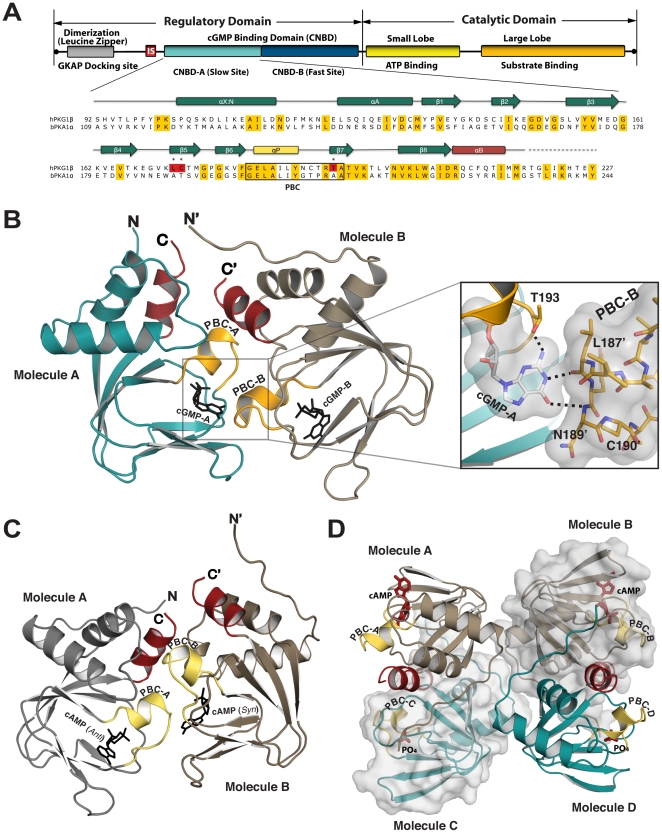
Domain organization and structures of the PKG Iβ (92–227). (A) Domain organization of PKG Iβ and sequence alignment with PKA RIα. 100% conserved residues are colored in yellow and PKG specific cGMP interacting residues are highlighted in red. (B) Overall structure of the PKG Iβ (92–227):cGMP complex showing the two molecules in the unit cell. The phosphate binding cassette (PBC) is shown in yellow, the αB helix in red, bound cGMPs are shown in black, and the N- and C-termini are labeled. Non-crystallographically related dimer contacts mediated by cGMP are shown as a zoom-in view on the right panel. In contrast to the solvent exposed cGMP in molecule B, the cGMP in molecule A (cGMP:A) is wedged between two molecules. The guanine ring of cGMP:A interacts with the tip of PBC of molecule B (PBC:B) through two hydrogen bonds as shown. Van der Waals surfaces of the bound cGMP:A and PBC:B are shown in gray. (C) Overall structure of the PKG1β (92–227):cAMP complex showing two molecules in the unit cell. (D) Overall structure of the partial apo showing four molecules in the unit cell. Bound cAMP and PO_4_ are labeled. All structure figures were generated using *PyMOL* (Delano Scientific).

The cGMP-dependent protein kinases (PKG) belong to the family of serine/threonine kinases and are one of the major intracellular receptors for cGMP. Mammals have two genes for PKG, *prkg1* and *prkg2*, which express PKG I and PKG II [1], [2], [3]. All PKGs have the same domain structure (Fig. 1A). An N-terminal leucine/isoleucine zipper is followed by an autoinhibitory sequence, which mediate homodimer formation and inhibit kinase activity, respectively. Next, two cyclic-nucleotide binding domains (CNBD-A and CNBD-B) are followed by the catalytic domain. In PKG I, the two CNBDs share approximately 37% amino acid sequence similarity but differ in their cGMP binding kinetics and cGMP analog specificities [4], [5]. CNBD-A provides a high-affinity (slow disassociation) site for cGMP whereas CNBD-B has a lower-affinity (fast disassociation) site. Differential splicing of the first 100 amino acids of PKG I mRNA produces PKG Iα and PKG Iβ isoforms, which have unique leucine/isoleucine zipper and autoinhibitory sequences but identical cGMP-binding and catalytic domains [2]. Binding of cGMP to the CNBDs is thought to induce a conformational change that activates the kinase by removing the autoinhibitory domain from the catalytic cleft, leaving the catalytic domain free to phosphorylate downstream substrates [6].

The overall architecture and molecular determinants for cAMP-specific CNBDs have been extensively studied using high-resolution crystal structures. These structures include the CNBDs from the *Escherichia coli* catabolite gene activator protein (CAP), cAMP-dependent protein kinase (PKA) and hyperpolarization-activated, cyclic nucleotide-modulated (HCN) channels [7], [8], [9]. However, in the absence of crystal structures, we know very little detail about cGMP-specific CNBDs and the molecular determinants for cGMP binding. To understand the overall architecture of the cGMP-binding domain and the molecular features required for cGMP binding, we determined crystal structures of the CNBD-A of human PKG I bound to cGMP, cAMP or in the absence of bound nucleotide. Our structures reveal that cGMP binds only in a *syn* configuration with a conserved threonine residue anchoring both cyclic phosphate and guanine moieties whereas cAMP binds in either *syn* or *anti* configuration with different sets of amino acid contacts. Surprisingly, our extensive isothermal titration calorimetry measurements show that CNBD-A binds both cGMP and cAMP with high affinity, showing only a two-fold preference for cGMP suggesting that other domains are required for the previously reported cyclic nucleotide selectivity.

## Results

### Structure determination and overall architecture

**Figure 2:**
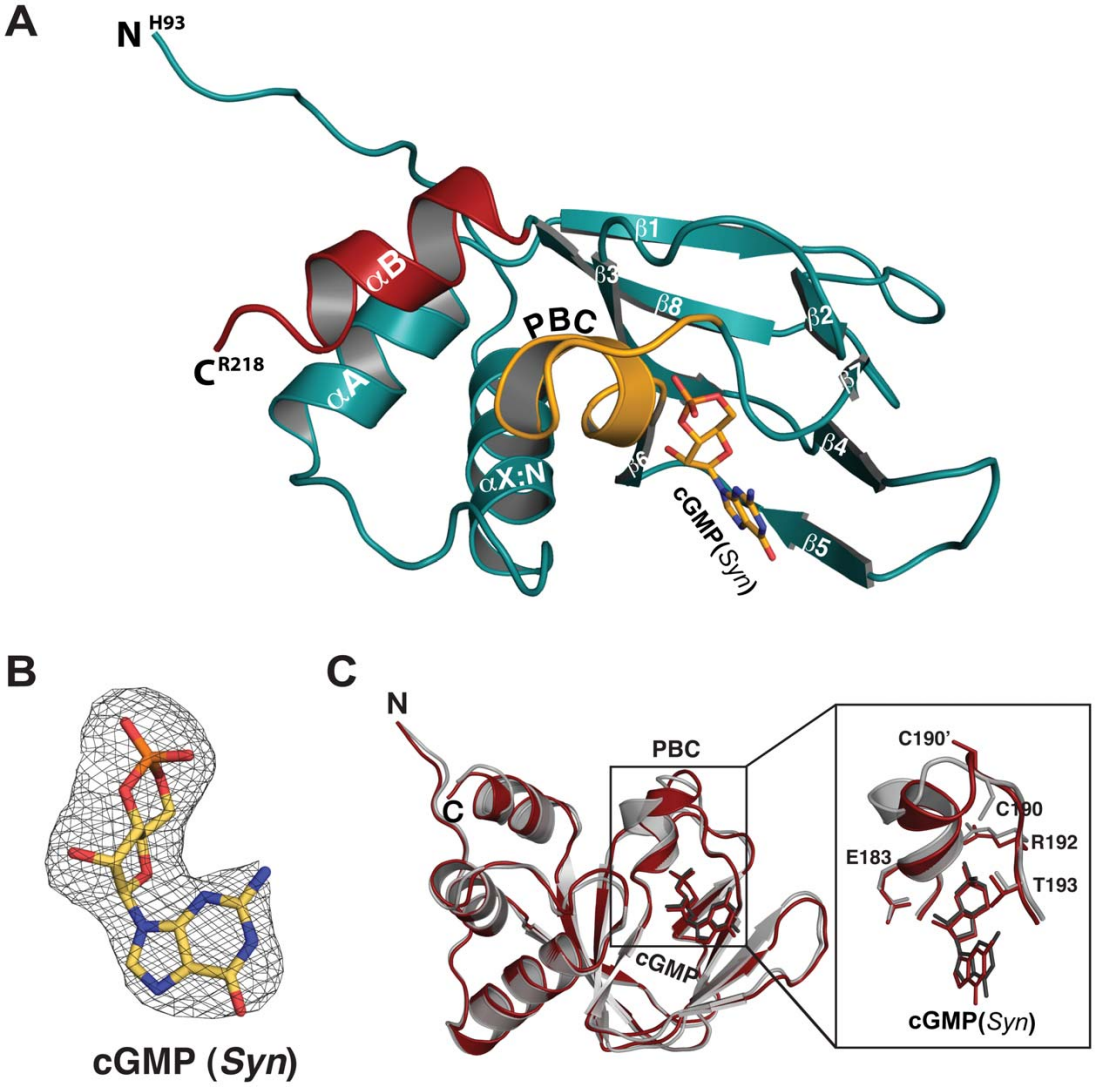
Structure of the PKG Iβ (92–227):cGMP complex. (A) Ribbon diagram of PKG Iβ (92–227):cGMP with its secondary structure elements labeled. (B) A *Fo-Fc* omit map showing the electron density of cGMP in *syn* conformation contoured at σ = 1.0. (C) Structural alignment of two mononers of PKG Iβ (92–227):cGMP complex with a zoom-in view of PBC on the right panel. Despite the crystal contacts, they align well with an rmsd of 0.87 Å for equivalent 127 Cα atoms.

The structure of PKG Iβ (92–227) in complex with cAMP was solved at 2.49 Å using a truncated model of PKA RIα (91–379) as a molecular replacement probe (PDB code: 1RGS)[8]. PKG Iβ:cGMP and partial-apo structures were subsequently solved at 2.9 Å and 2.75 Å respectively, using the fully refined structure of the PKG Iβ:cAMP complex as a molecular replacement model (Fig. 1 and Table 1). Refinement of the PKG Iβ:cGMP complex was carried out in PHENIX (dev-403) [10] using reference dihedral restraints derived from the higher resolution cAMP complex resulting a final model with R_work_ and R_free_ of 20.4% and 26.0%, respectively. The PKG Iβ:cAMP and PKG Iβ:cGMP complexes crystallized with two molecules per unit cell in a *P6_2_22* space group with over 75% solvent content. As predicted from its sequence similarity with the CNBDs from cAMP-effector proteins such as CAP, PKA and HCN [11], each molecule shows all of the predicted secondary elements, including: the two N-terminal helices, αX:N and αA helices; an 8-stranded anti-parallel β-barrel; and the B-helix at the C-terminus (Fig. 2A). The structure also contained a Phosphate Binding Cassette, (PBC), which is comprised of a short helix (P-helix) and loop and is situated between β6 and β7 strands (Fig. 2A). The crystallographic dimer is formed mainly by the bound cGMP, the helical tip of the PBC, and the αB-helix from one molecule (molecule B) fitting onto similar regions on the second molecule (molecule A) (Fig. 1B). While cGMP in molecule B (cGMP:B) is partially exposed to solvent, cGMP in molecule A (cGMP:A) is wedged between the two molecules and participates in crystallographic packing of the two molecules. Regardless of unique crystallographic contacts, they both bind each cGMP pocket in a *syn* configuration (Fig. 2B). cGMP:A interacts with the PBC of molecule B through two hydrogen bonds (Fig. 1B). Due to this contact, the tip of PBC in molecule B is distorted, tilting slightly toward the β-barrel. While the PKG Iβ:cAMP complex was crystallized in the same *P6_2_22* space group, with similar crystal parameters and contacts, cAMP:A does not form hydrogen bonds with molecule B and PBC:B shows no apparent contact induced structural changes (Fig. 2C).

**Table 1:** Data and refinement statistics.

Data set	cGMP bound	cAMP bound	Partial APO
Space group	*P6_2_22*	*P6_2_22*	*P4_3_*
Cell constants (Å)	a = b = 107, c = 171α = β = 90.0, γ = 120	a = b = 107, c = 169α = β = 90.0, γ = 120	a = b = 62.6, c = 202α = β = γ = 90.0
Wavelength (Å)	1.0	1.0	1.0
Resolution (Å)	50–2.9	50–2.49	45–2.75
Total/unique reflections	402498/13503	293611/20607	80424/19782
Average redundancy	29.8(20.2)	13.8(14.2)	4.1(4.1)
Completeness (%)	100(100)	98.7(99.6)	100(99.6)
<I>/<σ_I_>	21.2(2.10)	43.5(5.62)	31.2 (2.39)
R_sym_ ◊ (%)	13.5(n/a)	10.1(42.4)	5.9(46.4)
R_work_ || (%)	20.4	20.6	18.0
R_free_ ¶ (%)	26.0	23.0	25.1
Overall B value(Å^2^)	73.4	46.6	94.4
Rmsd bond length (Å)	0.010	0.014	0.005
Rmsd bond angle(°)	1.42	1.274	0.942

◊ *R*_sym_ = Σ*_h_*Σ*_i_*|*I*(*h*) - *I*(*h*)*_i_*|*I*Σ*_h_*Σ*_i_ I*(*h*)*_i_*, where *I*(*h*) is the mean intensity after rejections.

§ Numbers in parentheses correspond to the highest resolution shell of data, which were 2.98 to 2.90 for the cGMP, 2.53 to 2.49 Å for the cAMP and 2.85 to 2.75Å for APO.

|| *R*_work_ = Σ*_h_*||*F*_obs_(*h*)| -|*F*_calc_(*h*)||*I*Σ*_h_*|*F*_obs_(*h*)|; no *I*/σ cutoff was used during refinement.

¶ 5.0% of the observed intensities was excluded from refinement for cross validation purposes.

The partial-apo structure was crystallized in a *P4_3_* space group and contained four molecules per unit cell, showing a set of crystal contacts that are different from the PKG Iβ:cAMP and PKG Iβ:cGMP complexes (Fig. 1D and Table 1). Thus, in total we modeled eight CNBD-A molecules, from three crystal forms. Nearly the entire protein showed clear electron density, except for residues 219–227, which correspond to the C-helix that connects CNBD-A to CNBD-B. Despite the different crystal contacts, the overall structures of the eight CNBD-A molecules are very similar. Superposition of the cGMP-structure with the cAMP- and PO_4_-bound structures showed rmsd values of 1.2 Å and 0.98 Å for the Cα atoms of molecule A, and 0.65 Å and 0.44 Å for molecule B respectively. Due to the similarity between these structures, we will focus on the PKG Iβ:cGMP complex.

### Comparison with the CNBD-A of PKA RIα

**Figure 3:**
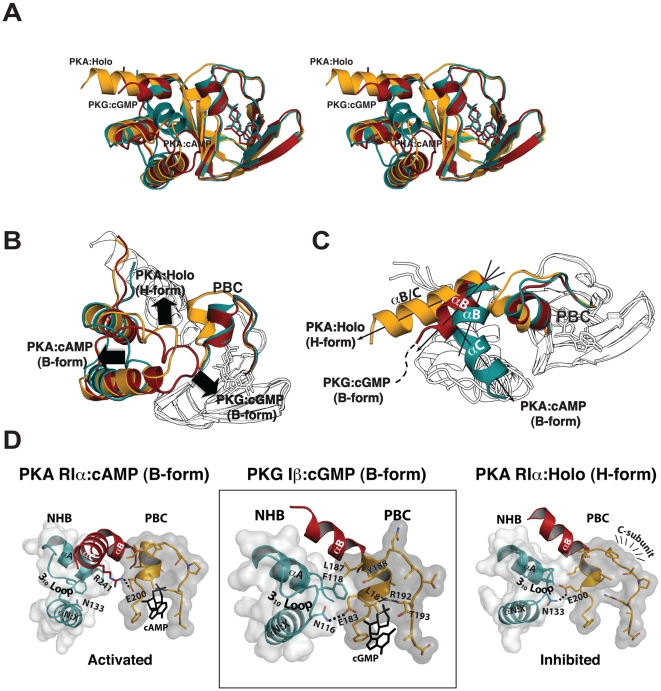
Structural comparison with cAMP-dependent protein kinase. (A) Stereoview of aligned CNBD-A from PKG and RIα of PKA. The PKG:cGMP complex is shown in the red. The PKA:cAMP (B-form) and PKA:Holo (H-form) are colored in cyan and yellow respectively. (B) A view showing differences in the conformation of the N-terminal helices, αX-N-3^10^ loop-αA with respect to the PBC. The PKG Iβ:cGMP complex is shown in red. The PKA:cAMP (B-form) and PKA:Holo (H-form) are colored cyan and yellow respectively. (C) A view highlighting the different conformations of the αB helix and the disordered αC helix in PKG versus PKA RIα. (D) The helical subdomains of PKG Iβ:cGMP, PKA RIα:cAMP (B-form), and PKA RIα:Holo (H-form) are shown.

Recent structural studies of PKA have shown that its CNBDs exist in two distinct conformations: a cAMP bound conformation that represents the activated state (B-form) [8] and a C-subunit bound conformation that represents the inactive state (H-form) [12], [13]. Conformational changes in response to cAMP or C-subunit binding involve rearrangement of the helical structures at the N- and C-terminus as well as the P-helix within the PBC. These helical rearrangements occur in relation to the structure of β-barrel, which remains essentially unchanged. Superimposition with the B and H forms of the PKA RIα CNBD-A reveals that the PKG Iβ CNBD-A:cGMP complex is in a conformation that more closely resembles the H-form of RIα, not the B-form (Fig. 3A). As seen in Fig. 3B, the helical subdomain of the PKG Iβ CNBD-A aligns better with the H-form of CNBD-A, which represents the C-subunit bound state. Like the H-form of RIα, the N-terminal helical bundle, consisting of the αX:N-α3_10_loop-αA helices, interacts with the PBC while the αB helix tilts up approximately 7° without engaging either motif (Fig 3C). In particular, the tip of α3_10_ loop reaches across the rigid β barrel making multiple contacts with PBC. The side chain of Asn116 forms a hydrogen bond with Glu183 which anchors the 2′ OH of the ribose (Fig. 3D). As in PKG Iβ CNBD-A, the H-form of PKA RIα shows a hydrogen bond between the corresponding asparagine and glutamate residues (Asn133 and Glu200 respectively) (right panel of Fig. 3D). In the B-form of RIα, Glu200 forms a salt bridge with Arg241 on the αC helix, which plays a major role in mediating PKA activation (left panel of Fig. 3D) [14]. Additional interactions that mediate the 3_10_-helix-PBC interaction include the carboxyl oxygen of Asn116 hydrogen bonding to the backbone amide of Phe118, whose side chain, in turn, makes a hydrophobic contact with Leu184, Tyr188 and Leu187 (middle panel of Fig. 3D).

### The cGMP binding pocket

**Figure 4:**
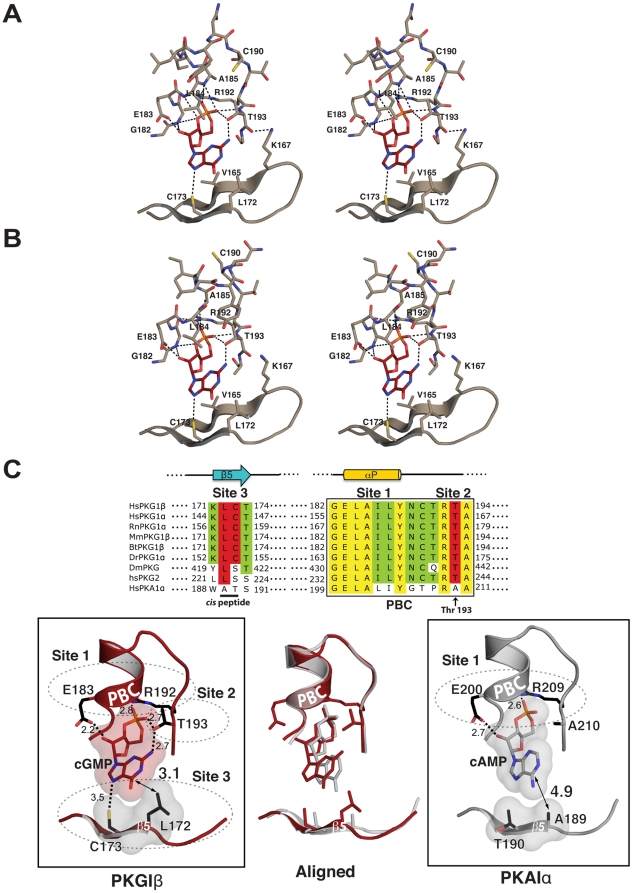
The cGMP binding pocket of PKG I and the cAMP binding pocket of PKA RIα. (A) and (B) Stereoview of the cGMP binding pockets showing their interactions with cGMP for molecule A and B respectively. (C) Aligned sequences at the cyclic nucleotide pockets are shown on top with each site labeled. 100% conserved residues are colored in yellow, over 60% conserved residues are colored in green, and the cGMP-specific interacting residues are shaded red; the cGMP binding pocket of PKG bound with cGMP is shown on the left, the cAMP pocket of PKA RIα with cAMP on the right and their alignment is shown in the middle panel. The Van der Waals surfaces of the bound cyclic nucleotides and the cis peptide forming residues are shown in surface representation.

Each cGMP binding site in the PKG Iβ:cGMP crystal shows a clear electron density for cGMP bound in a *syn* configuration (Fig. 2B), as previously predicted by mutation and other studies [4], [11], [15], [16]. Contacts between cGMP:A and PBC-B do not influence the overall interaction pattern of cGMP:A with the protein; the amino acid contacts with each cGMP are essentially the same (Figs. 4A and 4B). While the guanine rings are partially exposed to solvent for both molecules, the sugar-phosphates are buried in the pockets formed at the PBCs. The cGMP-binding site is comprised of three parts: the short P-helix together with conserved glutamate and arginine residues at the PBC which captures the sugar phosphate (Site 1); a key residue, Thr193 at the end of PBC that bridges the cyclic phosphate to the guanine ring (Site 2); and the β5-strand that provides a unique docking site for the guanine ring (Site 3). While the first site is shared with PKA, the other two sites are unique to PKG (Fig. 4C).

The first binding site consists of a positively charged pocket created by a cluster of unpaired backbone amides at the N-terminus of the P-helix and the side chain of Arg192 [8]. The exposed backbone amides of Gly182, Glu183, Leu184 and Ala185 of the P-helix together with the guanidinium group of Arg192, captures the cyclic phosphate through several hydrogen bonds and electrostatic interactions (Figs. 4, and Table 2). In addition, the side chain of Glu183 interacts with the 2′ OH of the ribose through a strong hydrogen bond.

**Table 2:** Protein-ligand distances.

		PKG I: cGMP complex		PKG I: cAMP complex				Partial Apo structure				PKA RIa:cAMP complex		
		cGMP-bound		Syn-cAMP bound		Anti-cAMP bound		cAMP-bound		PKG I:PO4				
Hydrogen Bonding Distances	**PKG I Residue Atom 1**	cGMP Atom	Distance from Atom 1 (Å)	cAMP Atom	Distance from Atom 1 (Å)	cAMP Atom	Distance from Atom 1 (Å)	cAMP Atom	Distance from Atom 1 (Å)	PO4 Atom	Distance from Atom 1 (Å)	**PKA Aligned Residue Atom 1**	cAMP Atom	Distance from Atom 1 (Å)
	**G182 N**	O2*	3.0	O2'	2.9	O2'	2.9	O2'	2.9	n/a	n/a	**G199N**	O2*	2.9
	**E183 OE1**	O2*	2.2	O2'	2.8	O2'	2.7	O2'	2.7	n/a	n/a	**E200 OE1**	O2*	2.6
	**A185 N**	O1P	3.2	O1P	2.9	O1P	2.9	O1P	2.9	n/a	n/a	**A202 N**	O1P	2.9
	**R192 NH1**	O1P	2.8	NH1	2.8	O1P	2.9	O1P	2.9	O4	2.8	**R209 NH1**	O1P	2.8
	**T193 N**	O2P	2.8	O2P	2.8	O2P	2.8	O2P	2.8	O1	2.7	**A210 N**	O2P	2.9
	**T193 OG1**	O2PN2	2.72.8	O2P	2.9	O2P	2.9	O2P	2.9	O1	2.5	**n/a**	n/a	n/a
VDW Distances	**V165 CG1**	C5	3.9	C5C8N7	3.83.93.9	C4	3.9	n/a	n/a	n/a	n/a	**n/a**	n/a	n/a
	**L172 CD**	N1O6	3.23.1	N6C6	3.93.7	N6	3.6	n/a	n/a	n/a	n/a	**n/a**	n/a	n/a
	**C173 SG**	N7	3.5	N7	3.6	C2	3.4	C2	3.4	n/a	n/a	**n/a**	n/a	n/a
	**M175 CE**	C8	3.9	C8	3.7	n/a	n/a	n/a	n/a	n/a	n/a	**n/a**	n/a	n/a

The second site, Thr193, is known to provide selectivity for cGMP [5]. This residue anchors cGMP through side-chain and backbone interactions. As seen in left panel of Fig. 4C, both the hydroxyl group and the carbonyl oxygen of Thr193 are within hydrogen-bonding distance to the 2-NH2 group of cGMP. In addition, the hydroxyl group of Thr193 interacts with the equatorial OP1 of cGMP, bridging the phosphate moiety to the guanine ring of cGMP. The side chains of neighboring residues, Leu184 and Cys190, help position the side chain orientation of Thr193 through hydrophobic packing with its Cγ atom. Thus, cGMP binding in the *syn* conformation is absolutely required for interaction with Thr193.

The third site is assembled by two consecutive residues, Leu172 and Cys173 on β5, and provides a docking site exclusively for the purine ring of cGMP (left panel of Fig. 4C). Leu172 and Cys173 are connected by an unusual non-proline cis-peptide bond, which orients their side chains toward the purine ring. While Leu172 makes a nonpolar contact with a carbonyl group at the C6 position of the guanine ring, Cys173 interacts with the unprotonated N7 of the guanine ring through an extended hydrogen bond. These interactions are only possible for cGMP bound in *syn* conformation. The interactions at sites 2 and 3 are essentially identical between the two molecules within the unit cell (Figs. 4A and 4B). Superposition with the PKA RIα:cAMP complex reveals differences in the relative orientation and amino acid composition of the site 3 forming residues (middle panel of Fig. 4C). Ala189 and Thr190 of RIα align with Leu172 and Cys173 of PKG Iβ, and despite forming cis-peptide bonds, they do not interact with cAMP (right panel of Fig. 4C). The β5 strand in RIα is located approximately 3 Å further away from the base than in PKG (middle panel of Fig. 4C).

Mutations of Thr193 have been shown to remove PKG's cGMP-binding selectivity, and the structures presented here are consistent with these results [5]. For example, mutation of this residue to alanine or valine resulted in a 27–29 fold increase in the amount of cGMP required for half-maximal kinase activation (*K_a_*), whereas substitution with serine required only 4 fold more cGMP. As seen in our structure, an alanine or valine substitution would completely abolish the interactions with the 2-NH_2_ group and the equatorial OP1 of cGMP, whereas a serine substitution would affect only the latter interaction, which explains the changes in cGMP affinity observed with each mutant. Notably, the cGMP binding site of CNG ion channels have a threonine at this position, and like PKG I substitution of this residue with alanine decreases cGMP sensitivity of the channel 30-fold without changing its cAMP sensitivity [17].

### Structure of cAMP-bound PKG Iβ CNBD-A

**Figure 5:**
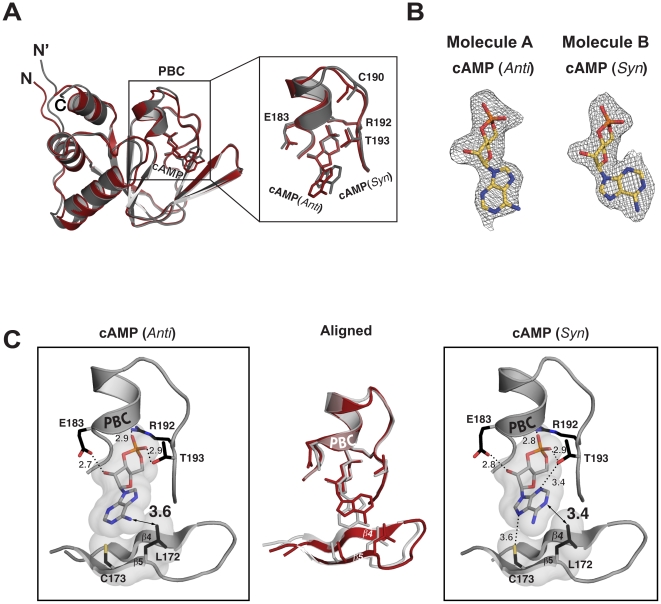
Structure of the PKG Iβ (92–227):cAMP complex. (A) Structural alignment of two mononers of PKG Iβ (92–227):cAMP complex. They align well with an rmsd of 0.74 Å for equivalent 126 Cα atoms. (B) A *Fo-Fc* omit map showing the electron density of cAMP in the *anti* and *syn* configuration contoured at σ = 1.0. (C) Showing two cGMP-binding pockets each with bound cAMP. cAMP in molecule is bound in a *anti* configuration (left panel) and an *syn* configuration in the other (right panel). Aligned structures are shown in the middle panel. The Van der Waals surfaces of the bound cAMPs and the cis peptide forming residues are shown in surface representation.

To gain additional insight into cyclic-nucleotide binding specificity, we determined the crystal structure of CNBD-A in the presence of cAMP. Despite its unique crystallization buffer conditions, the PKG Iβ:cAMP complex showed similar crystal parameters and contacts as the PKG Iβ:cGMP complex containing two molecules in the unit cell (Fig. 1C and Table 1). cAMP:A is similarly located at the interface between the two molecules, but makes no hydrogen bonds with molecule B. A surprising feature of the PKG Iβ (92–227):cAMP complex is that cAMP binds in two different conformations, *anti* in one molecule and *syn* in the other (Fig. 5). While the sugar phosphates share the same set of contacts with the protein as the PKG Iβ:cGMP complex at site 1, each purine ring of cAMP shows different contacts with the protein at sites 2 and 3, depending on its orientation. For example, the hydroxyl group of Thr193 at site 2 interacts with the unprotonated nitrogen at the 2-position through a weak hydrogen bond for the *syn-*configured cAMP whereas no such contact exists in the *anti*-configured cAMP. Leu172 at site 3 is within 3.6 Å and 3.4 Å for the *anti*- and *syn-* configured cAMP respectively (Fig. 5C). Cys173, makes a hydrogen bond with the unprotonated N7 of the *syn-*configured cAMP, whose distance is 3.6 Å, but no such contact exists for cAMP in the *anti* conformation (Fig. 5C). The side chains of Val165 and Met175 near site 3 come within 3.5–3.8 Å of the purine ring for *syn*-configured cAMP, but they are beyond van der Waals distance for *anti*-configured cAMP. Superposition of the two molecules at the PBC reveals that the differences in cAMP binding are mainly caused by the β4 and β5 strands moving away from the PBC to accommodate the extended conformation of the *anti*-configured cAMP (Fig. middle panel on Fig. 5C).

### Structure of Partial Apo PKG Iβ CNBD-A

**Figure 6:**
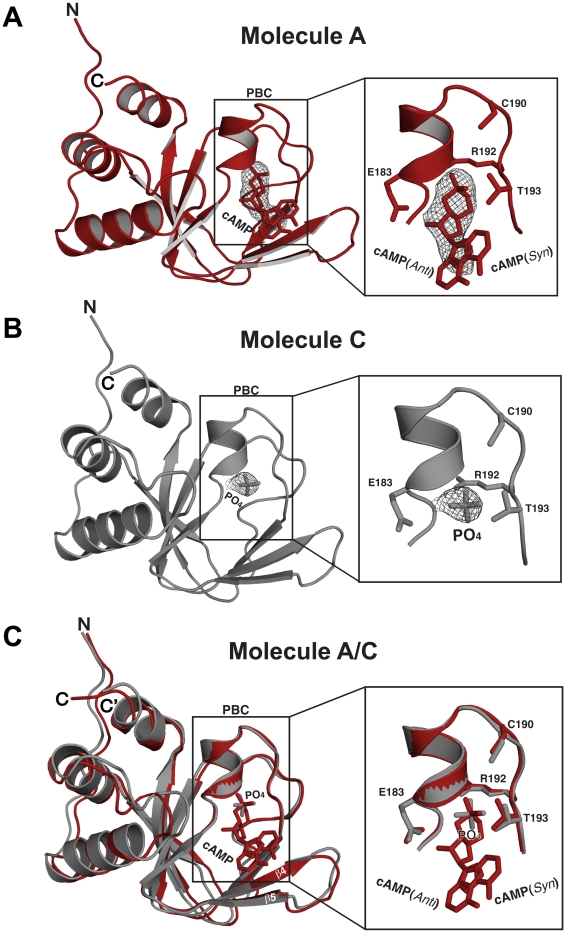
Structure of the PKG Iβ (92-227): Partial apo. (A) Structure of cAMP bound PKG Iβ (92–227) shown in cartoon representaion. The cAMP interacting residues and bound cAMP are shown in sticks. A *Fo-Fc* omit map showing the electron density of cAMP in *syn* and *anti* configuration contoured at σ = 1.0. (B) Structure of PO_4_ bound PKG Iβ (92–227). A *Fo-Fc* omit map showing the electron density of PO_4_ contoured at σ = 1.0. (C) Structural alignment of molecule A and C. Despite the crystal contacts and different ligands, they align well with an rmsd of 0.51 Å for equivalent 118 Cα atoms.

Our attempts to obtain crystal structures of an apo form of the CNBD-A yielded a partial apo structure, where two of the four molecules in the unit cell were bound by cAMP (CNBD-A:cAMP*_P43_*), which came from the *E. coli* cultures (Figs. 1D and 6A). Molecules without cAMP were bound by phosphate (CNBD-A:PO_4_), possibly due to high concentration of phosphate in the crystallizing solution (Figs. 1D and 6B). CNBD-A:PO_4_ superimposes well with CNBD-A:cAMP*_P43_*, except for the β4 and β5 strands (Fig. 6C). In the absence of cAMP, this region moves slightly away from the PBC resulting in a more open conformation.

In contrast to our CNBD-A:cAMP complex, where a full electron density was seen for cAMP bound in two different configurations, only partial electron density was seen for each bound cAMP in two of the four molecules in the CNBD-A:cAMP*_P43_* crystal, which accounts only for the sugar phosphate moiety and pyrimidine portion of the adenine ring (Figs. 6A and S1). This partial density can be explained by either *syn-* or *anti*-configured cAMP indiscriminately binding to the cGMP pocket, since either configuration of cAMP can be fitted to the partial electron density. The phosphate molecule, binds the same site as the cyclic phosphates, with the same set of interactions (Fig. 6C and Table 2).

### Cyclic nucleotide binding affinities of the PKG Iβ CNBD-A

**Figure 7:**
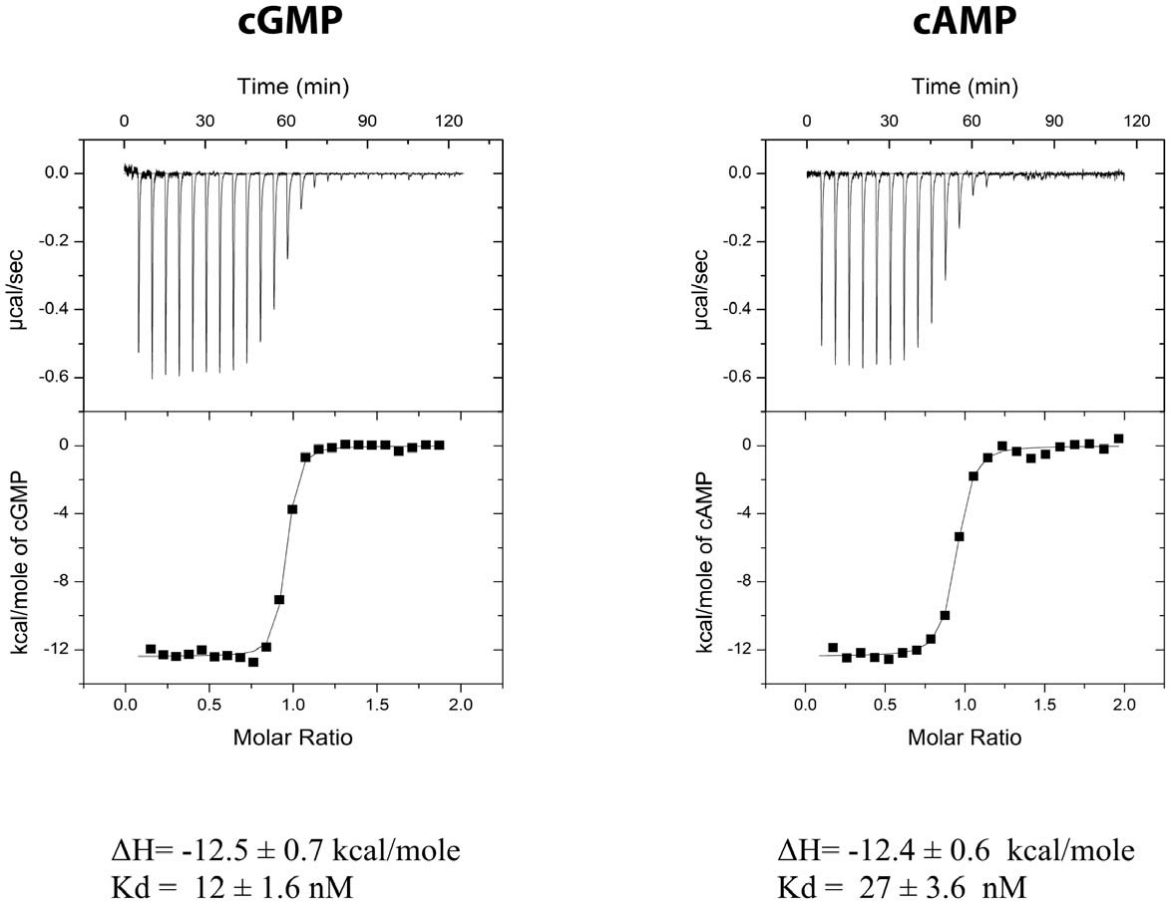
Isothermal titration calorimetry data for cGMP and cAMP binding to PKG Iβ (92–227). The calorimetric measurements for cGMP (panel a) or cAMP (panel b) binding were performed and analyzed as described in Material and Methods.

Next, we analyzed the binding characteristics of PKG Iβ CNBD-A to cyclic nucleotides using isothermal titration calorimetry (ITC). Our initial ITC measurements showed variable binding constants, indicating that the purified protein samples might contain different amounts of cAMP (verified by our partial apo structure). In order to remove cAMP carried over from *E. coli*, we denatured the protein in 6 M guanidine HCl and slowly refolded it, as described in Materials and Methods. ITC measurements were reproducible following denaturing and refolding. Unexpectedly, we found that CNBD-A binds both cGMP and cAMP with comparably high affinity (Fig. 7). Both cyclic nucleotides bind to the protein through strong enthalpy driving forces, with enthalpy values of −12.5 *versus* −12.4 kcal/mol at 30°C, suggesting that binding is driven by charge-charge interactions, most likely between the phosphate groups and the highly charged residues of the PBC. In contrast, the binding entropies are unfavorable (−4.7 cal/mol/K for cGMP and −6.1 cal/mol/K for cAMP at 30°C). Thus, the subtle difference in binding affinity (12 nM for cGMP and 27 nM for cAMP) is provided entirely by difference in the binding entropy terms, which suggests that the difference is due to hydrophobic interactions between the different purine bases and the protein.

## Discussion

While the basis for the cyclic-nucleotide specificity for PKG I has been previously studied, the exact molecular mechanism is not known. Because cGMP and cAMP are structurally different at only the 2-, 6-, and N1-positions of their purine rings, different amino acid contacts at these positions were proposed to mediate the specificity. Due to rotation around their glycosidic bonds, cyclic nucleotides exist in equilibrium between *syn* and *anti* conformations, with cGMP and cAMP favoring *syn* and *anti* conformations respectively [18], [19]. The cGMP-binding site of PKG and CNG channels has a threonine residue distinct from the cAMP receptors, and previous models based on the known structures of PKA and HCN channels have predicted that the hydroxyl group of these threonine residues interacts with the guanine 2-NH_2_ group of *syn*-cGMP through hydrogen bonds.

We attempted to crystallize several CNBD-A and CNBD-A/B domains of PKG I, based on the previously solved crystal structures of PKA RIα [8], [20]. So far, only the CNBD-A corresponding to PKG Iβ (92–227) has yielded good diffraction quality crystals. In all, we obtained three crystal forms and solved eight molecules of PKG Iβ (92–227), bound to a phosphate ion, cAMP or cGMP. Our structures explain some past biochemical observations on PKG I. One study demonstrated that intrachain disulphide bond formation between PKG Iα Cys117 and Cys195 (analogous to PKG Iβ Cys133 and Cys211) activates the kinase [21]. Consistent with this observation, the crystal structure of CNBD-A clearly shows that these residues are within the proper distance to form a disulphide bond upon oxidation (Fig. S2). These residues are located within the A- and B-helices, and in analogy to PKA, the B-helix is expected to form contacts with the catalytic domain. We speculate that disulphide bond formation between these residues alters the conformation of the B-helix such that it no longer forms a binding surface for the catalytic domain. Another study demonstrated that cGMP-binding protected full-length PKG Iα from cleavage by chymotrypsin at Met200 [22]. Our structure reveals that this methionine links the B-helix to the PBC through hydrophobic interactions. It appears that cGMP-induced stabilization of the PBC would provide a stable hydrophobic interaction surface for the methionine, providing a possible explanation for the observed protection.

**Figure 8:**
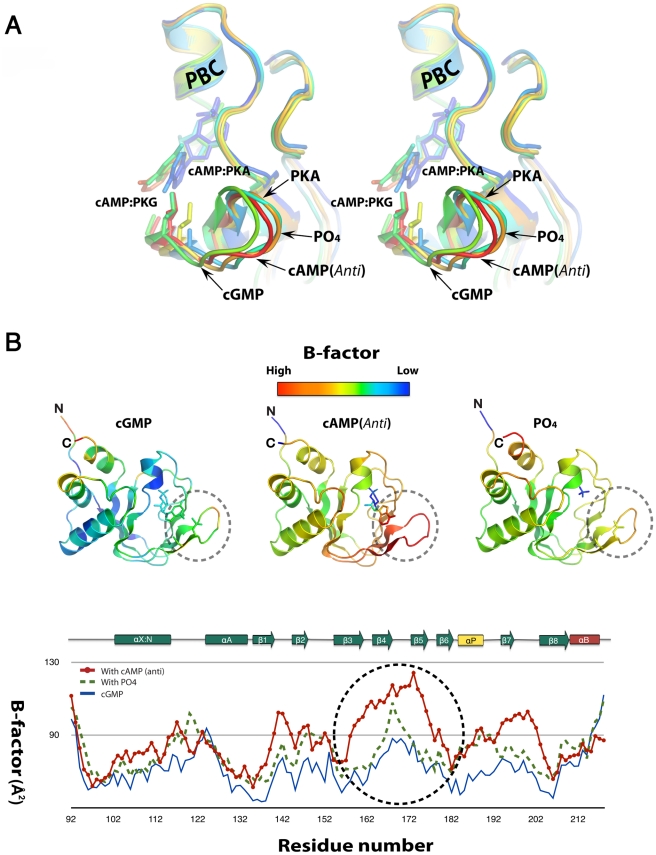
Structure and backbone B-factor comparison of the PKG Iβ:cGMP, PKG Iβ:cAMP*_P43_*, PKG Iβ:PO_4_ and PKA RIα:camp. (A) Stereo view of the cGMP binding pocket colored according to calculated B-factors. (B) The PKG Iβ:cGMP, PKG Iβ:cAMP*_P43_*, and PKG Iβ:PO_4_ are colored according to the B-factors of Cα atoms. The β4/β5 region of each structure is circled with a dotted line. B-factor plots of Cα atoms are shown below. The β4/β5 region is circled.

A direct comparison between the three structures of the PKG Iβ CNBD-A in the presence and absence of cyclic nucleotides, as well as with the homologous domain of PKA, provides a possible mechanism for cyclic nucleotide binding. In the absence of cyclic nucleotides, the conformation of CNBD-A is similar to the cyclic-nucleotide bound forms; with the exception of the β4/β5 strands which are in an open conformation with respect to PBC, as seen in the PO_4_ bound structure (Fig. 8A). The initial binding of cGMP, or cAMP, is likely to occur at site 1, mediated mainly by strong charge-charge interactions between the sugar phosphates and residues in the PBC. Both *syn-* or *anti*-configured cyclic nucleotides can bind equally at the site 1. Because the interaction pattern with the sugar phosphate is essentially identical for PKG and PKA, site 1 cannot provide the required cyclic-nucleotide selectivity. However, at site 2, only cGMP in a *syn* configuration positions its 2-NH_2_ group such that it can form a hydrogen bond with Thr193. Since a hydrogen atom replaces the 2-NH_2_ group in cAMP, no such interaction is possible, and cAMP binds the PKG CNBD-A in both *syn-* or *anti*-configurations (Table 2). Lastly, we found that the carbonyl at the 6-position and the unprotonated nitrogen at the 7-position of cGMP interact with the cis peptide forming residues, Leu172 and Cys173, resulting a “closed” conformation for the β4 and β5 strands. While there is only slight conformational differences within the β4/β5 region in our three CNBD-A structures, the temperature factors (B-factors) are noticeably different in this region (Fig. 8B). The CNBD-A bound with *syn*-configured cGMP shows the lowest B-factors, implying that interaction with the guanine ring is strongest at site 3 compared to other structures (Fig. 8B). In contrast, the structure with *anti*-configured cAMP shows the highest B-factors at this region, indicating that site 3 residues do not interact as strongly with the adenine ring. Although the corresponding residues in PKA, Ala189 and Thr190, are also connected by a cis-peptide bond, they do not interact with cAMP, and the β4 and β5 strands are further away from the nucleotide compared to PKG (Fig. 8A).

The cGMP-binding affinities for full-length PKG Iα and PKG Iβ, as well as their isolated regulatory domains, have been reported [11], [23], [24], [25], [26]. This report provides *K_d_* measurements of the isolated PKG Iβ CNBD-A for both cGMP and cAMP. While the *K_d_* for cGMP is somewhat similar to the previously reported values for full-length PKG Iα [24], the affinity for cAMP is remarkably high, being only a two-fold weaker than the value measured for cGMP (Fig. 7). This small difference in binding affinity was unexpected as full-length PKG Iα has a 100-fold lower affinity for cAMP than cGMP. Our results may be explained in a number of ways. Most likely, our results reflect the fact that we are using a truncated protein and the binding affinities observed for the full-length protein are caused by allosteric interactions between CNBD-A other regions of the R-domain, such as the leucine/isoleucine zipper, the autoinhibitory sequence and/or CNBD-B. In fact, previous research has shown that the N-terminal leucine/isoleucine zipper and/or autoinhibitory regions modulate cGMP-affinity of the cyclic nucleotide binding pockets [27], [28], [29], [30]. In addition, our CNBD-A construct lacks regions of the R-domain that are expected to interact with the cGMP-binding pocket. Indeed, unlike what is seen in other cGMP- and cAMP-pockets, our structures show that the nucleotides are partially exposed to solvent, whereas in PKA RIα “capping” residues increase cAMP affinity by covering the cyclic nucleotide binding pocket. Using models of PKG I CNBD-A/B domain, constructed using crystal structures of the R-subunit of PKA, we find that the C-helix of CNBD-A or the A-helix of CNBD-B may position near the solvent exposed side of the binding pocket. Since the cGMP affinity of PKG I CNBD-A is similar to reported values for full-length PKG Iα, we speculate that these contacts lower the affinity for cAMP, thus providing sufficient affinity differential for cyclic nucleotide selectivity.

### Conclusion

Despite the high degree of similarity between PKA and PKG, our structures reveal that the molecular interactions that mediate cyclic nucleotide binding are distinct from PKA. These interactions may explain reported differences between the regulation of PKG and PKA, such as the reversed order of the high and low affinity CNBDs and differences in cyclic nucleotide induced conformational changes, as revealed by small angle X-ray scattering [5], [31]. The finding that the cGMP-bound PKG looks structurally more like C-subunit bound PKA RIα was unexpected, as was the interaction between Leu172/Cys173 and the guanine base. The small difference between cGMP and cAMP affinity was also unexpected, but not completely surprising since our construct represents a single domain and domain-domain interactions have been previously shown to modulate cGMP affinity [32]. We are currently working to extend our structural analysis of PKG I to include the CNBD-B domain; these studies should reveal additional molecular contacts that modulate cyclic nucleotide affinity. Because most effects of cGMP elevating drugs, such as organic nitrates and phosphodiesterase inhibitors, are mediated by PKG, direct activators of PKG could provide novel approaches to treat a wide array of hypertensive diseases. The structures presented here will be useful for designing such reagents.

## Materials and Methods

### Protein Expression and Purification

A DNA sequence encoding Human PKG Iβ (92–227) was cloned into pQTEV [33]. The protein was produced in BL21 (DE3) *E. coli* which were grown at 37°C until OD_600_ of 0.6 then induced with 0.4 mM IPTG. The cultures were grown for an additional 18 hours at 18°C. Cells were suspended in 50 mM Tris, 150 mM NaCl, 1 mM DTT (pH 7.9) and lysed using a cell disruptor (Constant Systems). His-tagged PKG Iβ (92–227) was purified with a BioRad IMAC resin on a Bio-Rad Profinia™ purification system. The protein was eluted with cell suspension buffer containing 250 mM imidazole. The sample was incubated with 1.0 mg/ml TEV protease at 4°C for 48 hours to remove the His-tag. The protein was purified further with a Q sepharose HP followed by gel filtration on a Hi-load 16/60 Superdex-75 column (GE Healthcare) in 25 mM Tris-HCl, pH 8.0, NaCl 150 mM and 1 mM TCEP-HCl.

### Crystallization

For the crystallization of the partial apo crystals, the protein sample was concentrated to 20 mg/ml using a 10 kDa cutoff Amicon Ultra (Millipore). The partial apo crystals were obtained using the vapor diffusion method in 1.4 M sodium/potassium phosphate (pH 5.6) at 22°C. Crystal optimization was done using an Orxy6™ robot (Douglas Instruments LTD). The bipyramidal crystals appeared in 1.4 M sodium/potassium phosphate (pH 8.1) at 22°C in 2 days. Co-crystallization with cGMP was accomplished by adding cGMP (Aral Biosynthetics) to a final concentration of 5 mM to the purified protein sample, which was then concentrated to 33 mg/ml using a 10 kDa cutoff Amicon Ultra (Millipore). The crystals of the PKG Iβ:cGMP complex were obtained using the vapor diffusion method in 0.1 M sodium malonate (pH 5.0), 12% PEG 3350 at 4°C. Similarly, co-crystallization with cAMP was accomplished by adding cAMP to a final concentration of 5 mM to the protein sample, which was concentrated with a 10 kDa cutoff Amicon Ultra (Millipore) to 17 mg/ml. The PKG Iβ:cAMP complex crystals were obtained using the vapor diffusion method in 1.4 M sodium/potassium phosphate (pH 5.6) at 4°C.

All crystals were transferred to a cryoprotectant solution (25% glycerol) and flash cooled in liquid nitrogen. X-ray diffraction data were collected at beamline 8.2.1 (Advanced Light Source, Berkeley, CA, USA). Diffraction data were processed and scaled using HKL2000, resulting in acceptable data set with satisfactory summary statistics (Table 1).

The crystal structure of PKG Iβ (92–227):cAMP was determined by molecular replacement using a truncated model of PKA RIα (91–379) (PDB: 1RGS) as a molecular replacement probe [8]. Subsequent phasing, density modification and model building were carried out with phenix.autosol [34]. The resulting model was manually completed in Coot [35] and restrained-structure-refinement implementing TLS refinement [36] resulted in cAMP model with R_work_ and R_free_ of 20.6% and 23.0% respectively. Refinement of the 2.9 Å PKG Iβ(92–227):cGMP complex was carried out in PHENIX (dev-403) [10] using reference dihedral restraints derived from the higher resolution cAMP complex, as described in the following section. Use of the higher resolution reference model in refinement improved the R and R-free values, as well as MolProbity validation criteria, resulting a final model with R_work_ and R_free_ of 20.4% and 26.0%, respectively [37]. For all of the *Fo-Fc* omit maps shown in the figures, we generated simulated annealing omit maps, omitting a region with a border of 2 Å around each ligand as described in Terwilliger et al.[38].

### Reference model refinement in phenix.refine

To improve refinement stability and associated model quality in low resolution refinement, the cGMP and partial apo structures were refined with *phenix.refine* using dihedral restraints obtained from the higher resolution PKG Iβ:cAMP structure. Dihedral restraints obtained from the reference model were imposed on the working model if the absolute angular deviation fell within a user-defined threshold. For this refinement, a threshold value of 15° was used. These restraints served to direct the overall topology of the model while avoiding unjustified bias to the high-resolution model. The refinement scheme is similar in concept to non-crystallographic symmetry restraints adopted in SHELXL and the deformable elastic network approach introduced in the following reference [39].

### Isothermal Titration calorimetry

To remove residual cAMP, all samples were denatured by incubating in 6 M guanidine HCl for 24 h at 4°C, then renatured by step-wise dialysis against first 2 M and then 0.5 M guanidine HCl over 48 h. The samples were then purified in 10 mM Tris (pH 8.0) and 150 mM NaCl on a Hi-load 16/60 Superdex 75 column (GE Healthcare). The calorimetric measurements for cAMP and of cGMP binding to PKG Iβ (92–227) were carried out using a VP-ITC calorimeter (MicroCal LLC, Northampton, MA). The protein was placed in the sample cell at a concentration of 15 µM in the column buffer. Cyclic nucleotides were placed in the injection syringe at a concentration of 250 µM. The injection volume was 5 μl. The data was processed using the Origin software with a manufacturer-supplied custom-addon ITC sub-routine. The reported results were repeated in at least duplicate.

### Protein data bank accession codes

The coordinates for the structures described herein have been deposited in the Protein Data Bank under the accession codes 3OD0, 3OCP and 3OGJ for PKG Iβ:cGMP, PKG Iβ:cAMP and the partial apo structures, respectively.

## Supporting Information

Figure S1 **A** ***Fo-Fc*** **omit map of cAMP and PO_4_ in the PKG Iβ (92–227): Partial apo structure.**
A *Fo-Fc* omit map showing the electron density of cAMP and PO_4_ along with the omitted region shown in mesh. A simulated annealing omit map was generated, omitting a region with a border of 2 Å around the bound cAMP and PO_4_.
(TIF)

Figure S2 **A view showing Cys133 and Cys211 of PKG1 β CNBD-A.**
(TIF)
