# Supplementary figures and images for: Co-Crystal Structures of PKG Iβ (92–227) with cGMP and cAMP Reveal the Molecular Details of Cyclic-Nucleotide Binding
Source: PLoS One. 2011 Apr 19;6(4):e18413. doi: 10.1371/journal.pone.0018413 (PMC3080414; doi:10.1371/journal.pone.0018413)

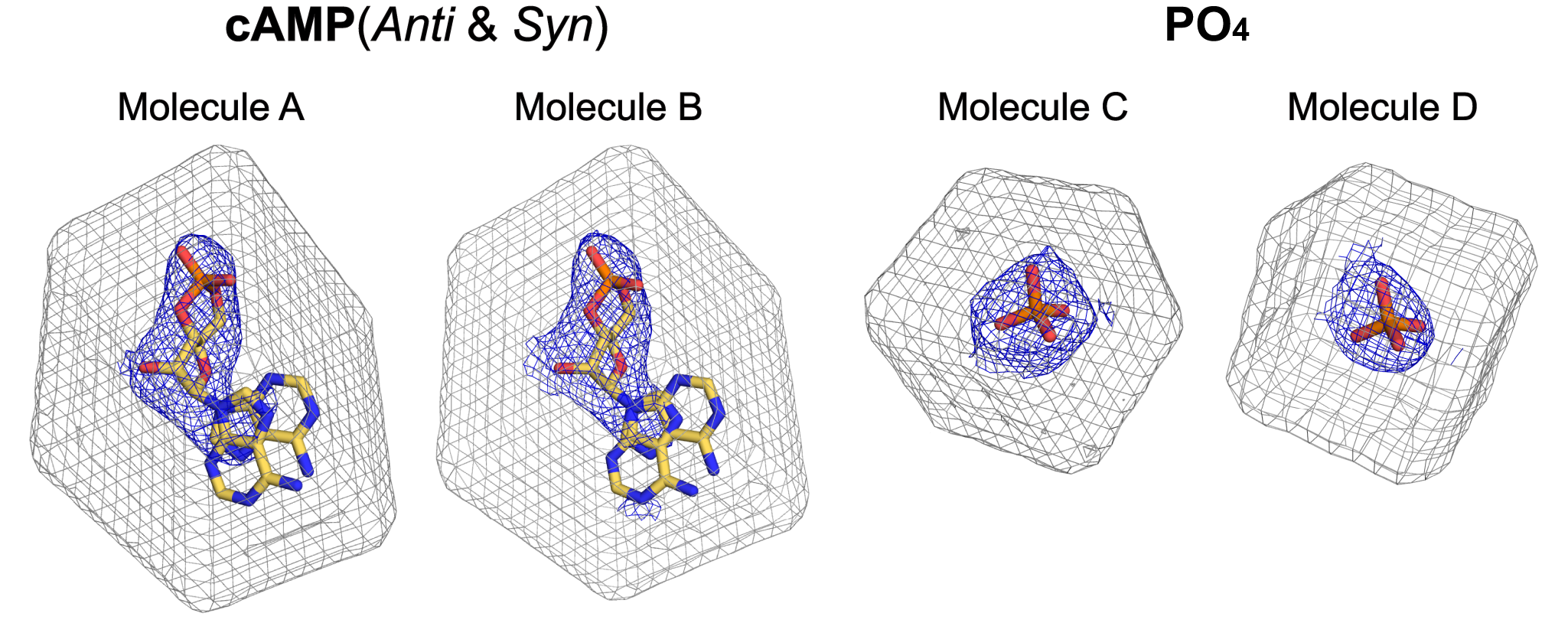

Supplement: Figure S1 — A Fo-Fc omit map of cAMP and PO4 in the PKG Iβ (92–227): Partial apo structure. A Fo-Fc omit map showing the electron density of cAMP and PO4 along with the omitted region shown in mesh. A simulated annealing omit map was generated, omitting a region with a border of 2 Å around the bound cAMP and PO4. (TIF) [file pone.0018413.s001.tif]

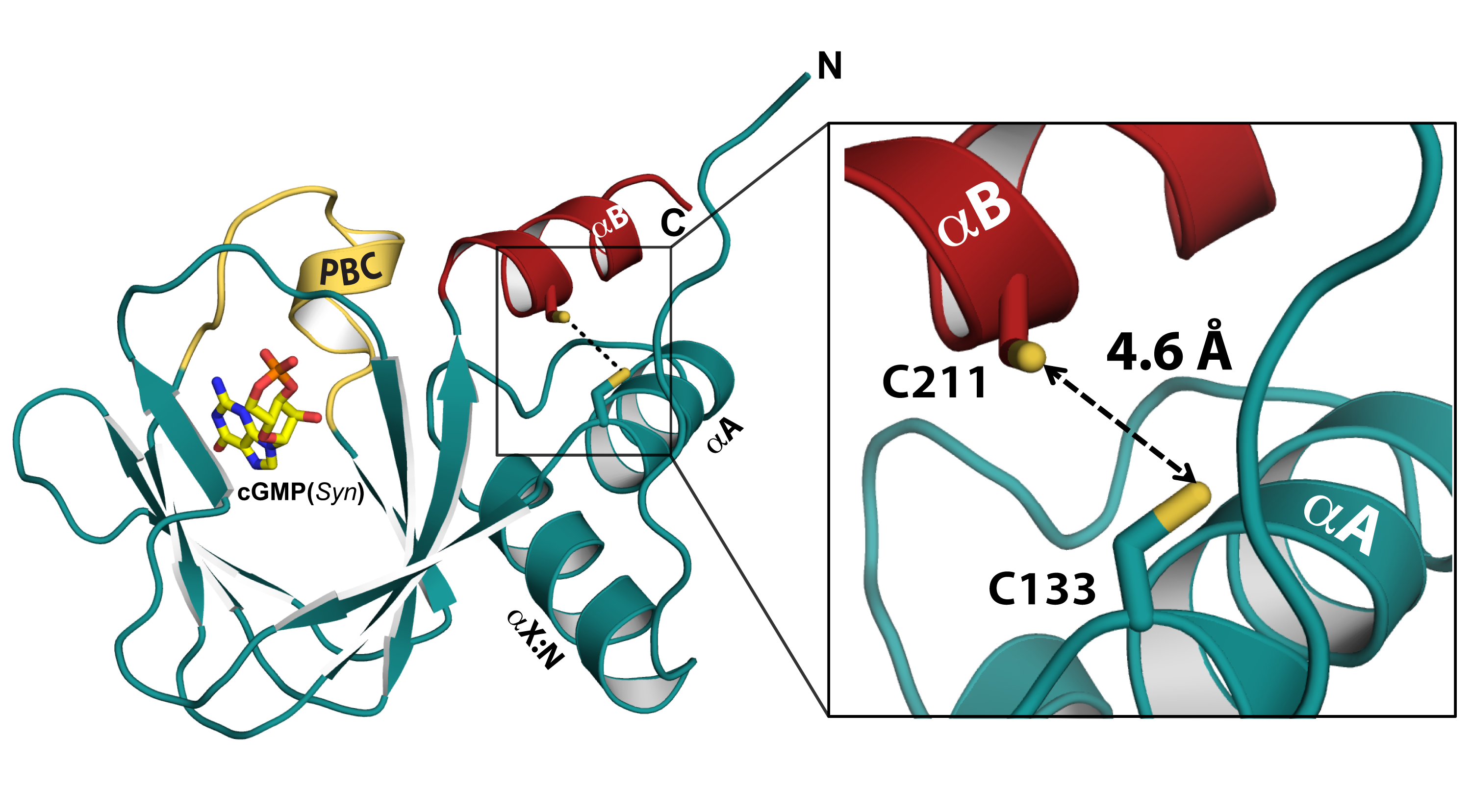

Supplement: Figure S2 — A view showing Cys133 and Cys211 of PKG1 β CNBD-A. (TIF) [file pone.0018413.s002.tif]
